# Formation of single-dominant-species patches of *Dicranopteris dichotoma* primarily influenced by understory light intensity

**DOI:** 10.3389/fpls.2024.1444371

**Published:** 2024-11-11

**Authors:** Dubin Dong, Jiali Tong, Liang Liao, Lita Yi, Wende Yan, Fei Yu

**Affiliations:** ^1^ College of Life Science and Technology, Central South University of Forestry and Technology, Changsha, China; ^2^ School of Forestry and Biotechnology, Zhejiang Agriculture and Forestry University, Hangzhou, China

**Keywords:** *Dicranopteris dichotoma*, subtropics, clonal plant, climate, community characteristics, soil

## Abstract

**Introduction:**

The *Dicranopteris dichotoma* fern community plays vital roles in nutrient sequestration, succession regulation, and ecological threshold control. However, the mechanisms underlying the formation of the *D. dichotoma*–dominant community remain unclear.

**Methods:**

This study established four different community types to investigate the effects of environmental factors on the formation of a *D. dichotoma*–dominant community.

**Results:**

We found that climate was the primary factor affecting the formation of patches dominated by *D. dichotoma* at the regional scale. Specifically, higher annual mean temperature and annual mean precipitation were associated with larger single-dominant-species patches of *D. dichotoma*. Understory light intensity was the major factor affecting the formation of the *D. dichotoma* community at the community scale. Light intensity ranging from 200 to 500 µmol·m⁻²·s⁻¹ was most conducive to the development of a large *D. dichotoma* community. Additionally, understory light intensity enhanced the importance value of *D. dichotoma* in the herb community by decreasing its biomass proportion of support modules and increasing its biomass proportion of photosynthetic and reproductive modules. Soil properties and *D. dichotoma* characteristics showed interactions with each other. Acidic red-yellow soil was most suitable for the formation of single-dominant-species patches of *D. dichotoma*, and the growth of *D. dichotoma* further decreased the soil pH. Soil total phosphorus content was identified as a limiting factor for formation of the *D. dichotoma* community.

**Discussion:**

In summary, the formation of single-dominant-species patches of *D. dichotoma* is mainly influenced by a combination of climate, community, and soil.

## 1 Introduction

As important components of tropical and subtropical ecosystems, ferns play crucial roles in microclimate maintenance, nutrient cycling, and energy circulation (Mehltreter et al., 2010; Tůma et al., 2012; Dalling et al., 2024). *Dicranopteris dichotoma* is a pioneer fern in subtropical low mountainous and hilly terrains. *D. dichotoma* thrives with a high light intensity and exhibits characteristics of high-temperature tolerance, acidity resistance, and adaptability to poor soils and drought conditions (Chen et al., 2016a; Wang et al., 2022). In degraded forests, *D. dichotoma* reproduces via sexual reproduction (spores) during the early colonization stage and then rapidly forms a single-dominant-species community via vegetative reproduction (clones) (Zhu et al., 2016). Therefore, the *D. dichotoma* single-dominant-species community is commonly found under pioneer tree species such as *Pinus massoniana* in the subtropical region of China (Qian and Chen, 1959).

*D. dichotoma* is primarily a clonal plant, and rhizomes are its main clonal reproductive organs. The rhizomes of *D. dichotoma* grow horizontally in the 0–4 cm soil depth, bearing leaf buds, adventitious roots, and new rhizome branches. These rhizome branches interweave to form a network that, together with the adventitious roots, envelops the detritus and soil, creating a “root sieve” with a thickness up to 10 cm (Zhang et al., 2010a). The leaf buds develop into fronds, forming a dense aerial layer. Due to the dense aerial layer and strong filtering effect of the root sieve, a significant number of seeds are prevented from entering the soil. Additionally, *D. dichotoma* inhibits the germination and growth of the seeds of other plant species through allelopathy (Juma et al., 2019). Various biological and chemical properties have been proposed as crucial factors in the formation and persistence of single-dominant-species patches of *D. dichotoma* in the last few decades (Yang et al., 2021).

Previously, the *D. dichotoma* community was considered a negative factor in the regeneration of the evergreen broadleaf forest posing potential risks such as forest fires, reduced nutrient cycling rates, and diminished plant diversity (Zhang et al., 2010a). However, recent studies have demonstrated the contribution of herbaceous layers to ecosystem nutrient cycling and accumulation, leading to reevaluation of the potential beneficial roles of *D. dichotoma* in ecosystem restoration and community succession. Indeed, recent studies have found that *D. dichotoma* slows the regeneration rate of the subtropical forest through “ecological filtering” but does not change the direction of succession (Liu et al., 2021). These findings indicate that *D. dichotoma* might slow down the succession process and prolong the soil recovery time. Therefore, *D. dichotoma* appears to play vital roles in nutrient sequestration, succession regulation, and ecological threshold control (Yang et al., 2021).

Nevertheless, the detailed formation mechanism of the single-dominant community of *D. dichotoma* in subtropical forests remains unclear. It is generally agreed that a lack of light in the understory is a crucial factor restricting the growth of *D. dichotoma*. This observation is aligned with the principle of heliotic plant withdrawal from the community. To address this question, we conducted controlled greenhouse experiments to evaluate the physiological characteristics of potted *D. dichotoma* under various light intensities. Additionally, a semi-field experiment was conducted by placing potted *D. dichotoma* under shrubs, *P. massoniana*, and evergreen broadleaved forest species. The results were not consistent with the previously established hypothesis. Instead, the experiments revealed that *D. dichotoma* exhibits a preference for medium-intensity light (300–800 µmol·m^−2^·s^−1^) and is intolerant to strong (>1800 µmol·m^−2^·s^−1^) and weak (<100 µmol·m^−2^·s^−1^) light intensities (Wang et al., 2021; Liao et al., 2023).

The asexual reproduction mode of *D. dichotoma* is closely linked to rhizome growth and leaf bud development. The rhizomes can branch indefinitely, and the fronds grow in a lush manner in moist and fertile soil, reaching heights of over 1 m (Wang et al., 2022). In contrast, in poor and arid soil conditions, the growth of the rhizomes ceases and the fronds are sparse and stunted, with heights reaching only 10–15 cm (Liu, 2017). Therefore, soil physicochemical properties, particularly the soil nutrient content, greatly impact *D. dichotoma* clonal reproduction (Lin, 2020; Bai et al., 2020; Wang et al., 2021). In turn, the *D. dichotoma* community can also impact the soil’s physical and chemical properties (Wan et al., 2014; Chen et al., 2016b; Lyu et al., 2019). However, previous studies have primarily involved small-scale field or pot planting experiments, with only a few large-scale field studies on *D. dichotoma* conducted to date. Consequently, our understanding of the interaction mechanisms between *D. dichotoma* community formation and environmental factors remain limited.

To fill this gap, in the present study, we investigated the associations of climate, community, soil, and topography characteristics in four common subtropical communities with the clonal dispersal traits of *D. dichotoma* in these communities. We established the following hypotheses: (H1) at the regional scale, the formation of large single-dominant-species patches of *D. dichotoma* is primarily influenced by climate; (H2) moderate levels of understory light (300–800 µmol·m⁻²·s⁻¹) are most favorable for the formation of the *D. dichotoma* community; and (H3) higher soil nitrogen and phosphorus nutrient contents promote the growth of *D. dichotoma*.

## 2 Materials and methods

### 2.1 Study areas and experimental design

**Figure 1:**
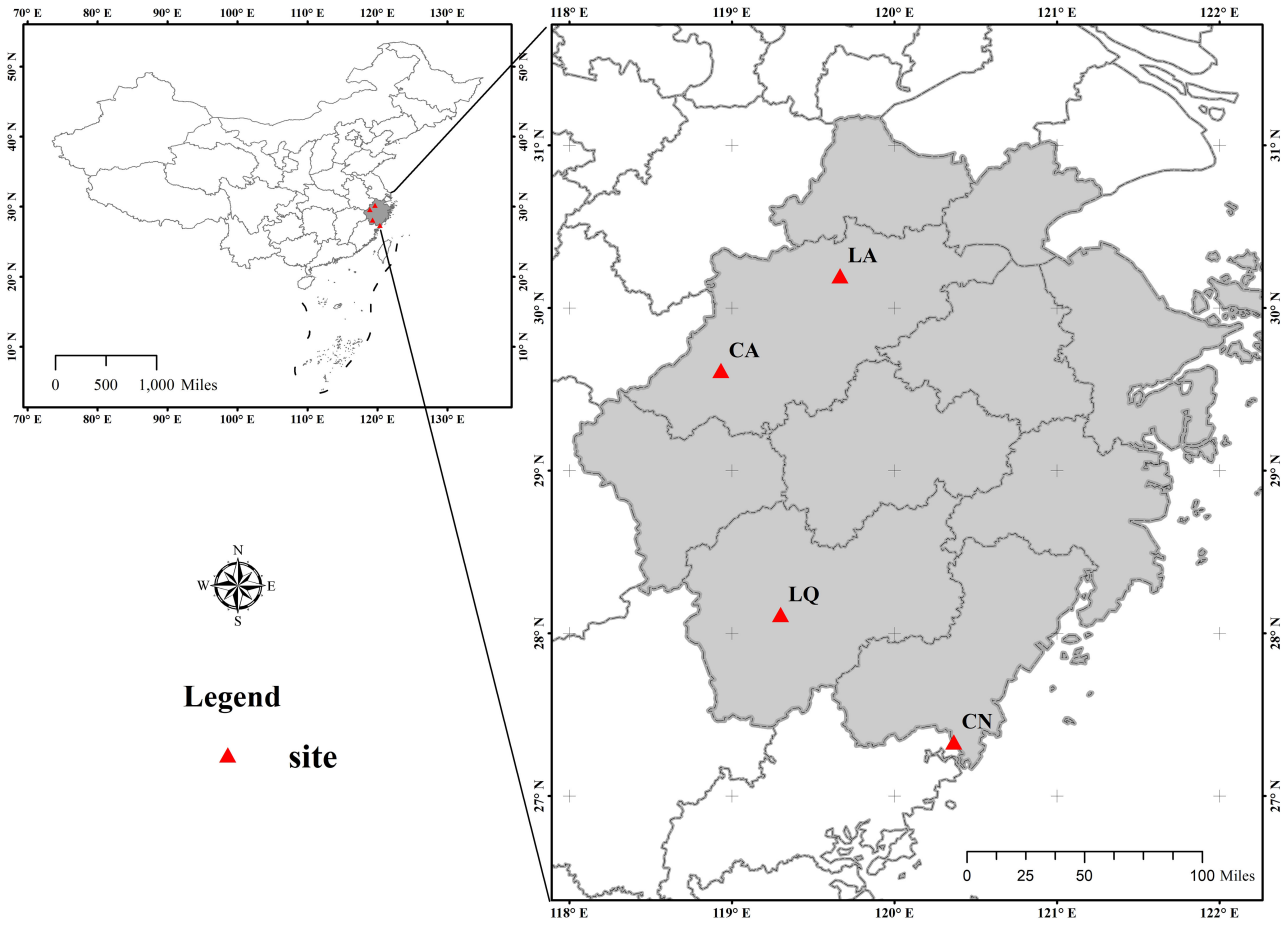
Map showing the location of the study areas (red triangles). LA, Lin’an District in Hangzhou; CA, Chun’an County in Hangzhou; LQ, Longquan City in Lishui; CN, Cangnan County in Wenzhou. This map is based on the standard map No. GS (2024) 0650, downloaded from the Standard Map Service Website of the Ministry of Natural Resources, China. The base map was not modified.

Communities dominated by *D. dichotoma* in the herb layer were selected as the study stands. The stands were located from north to south across multiple sites in Zhejiang Province, encompassing Lin’an District in Hangzhou (LA), Chun’an County in Hangzhou (CA), Longquan City in Lishui (LQ), and Cangnan County in Wenzhou (CN), as shown in **Figure 1**. The study region has a mid-subtropical monsoon climate characterized by distinct seasons with prolonged winters and summers and shorter springs and autumns. The predominant soil type is red-yellow loam. The annual mean temperature and annual mean precipitation increased progressively across each study site, while the annual mean sunshine hours decreased accordingly in a north-to-south direction (**Table 1**). All study sites were located on slopes or gentle slopes at elevations of 200–300 m (**Table 2**). The community types include grassy slopes, coniferous forests, coniferous and broadleaved mixed forests, and evergreen broadleaved forests, all of which are secondary communities (the community information of the study sites was detailed in **Tables 2**, **3**).

**Table 1:** Location and climate information of study areas.

Study sites	Latitude	Longitude	Annual mean temperature (°C)	Annual mean precipitation (mm)	Annual mean sunshine hours (h)
**LA**	30°12’52’’ N-30°13’1’’ N	119°40’14’’ E-119°40’22’’ E	15.79	1315	1677
**CA**	29°37’18’’ N- 29°37’’ 20’’ N	118°56’7’’ E-118°57’9’’ E	16.78	1431	1684
**LQ-AR**	28°7’6’’ N- 28°7’6’’ N	119°4’9’’ E-119°18’28’’ E	16.44	1587	1574
**LQ-LJ**	28°7’6’’ N- 28°7’6’’ N	119°4’9’’ E-119°18’28’’ E	16.44	1587	1574
**CN**	27° 20’42’’ N-27°59’54’’ N	120°22’14’’ E-120°22’25’’ E	18.67	1705	1583

LA, Lin’an District in Hangzhou; CA, Chun’an County in Hangzhou; LQ-AR, An’ren Township of Longquan City in Lishui; LQ-LJ, Lanju Township of Longquan City in Lishui; CN, Cangnan County in Wenzhou.

**Table 2:** Community information of study areas.

Study sites	Plot	Elevation (m)	Slope gradient (°)	Aspect	Community types	Canopy closure (%)	Dominant tree	Average tree height (m)	Average diameter at breast height (cm)	Average tree age (a)	Dominant shrub
**LA**	1	240	38	South, 183	Conifer-broadleaf forests	0.88	*Pinus massoniana; Schima superba*	5.68	11.89	15	*Camellia japonica*, *Loropetalum chinense*,
	2	229	20	Northwest, 329	Conifer-broadleaf forests	0.87	*Pinus massoniana; Schima superba*	7.76	6.80	15	*Camellia japonica, Eurya japonica*
	3	203	36	Southeast, 116	Conifer-broadleaf forests	0.83	*Pinus massoniana; Schima superba*	5.88	7.40	15	*Camellia japonica*, *Rhododendron simisii*
**CA**	1	184	34	Southwest, 234	Evergreen broadleaved forests	0.65	*Castanopsis sclerophylla*	6.74	11.17	20	*Camellia japonica*
	2	188	23	South, 196	Evergreen broadleaved forests	0.73	*Castanopsis sclerophylla*	7.15	12.67	20	*Loropetalum chinense*
	3	198	29	Southwest, 215	Evergreen broadleaved forests	0.87	*Castanopsis sclerophylla*	7.36	12.13	20	*Loropetalum chinense*
**LQ-AR**	4	254	14	Southeast, 122	Evergreen broadleaved forests	0.63	*Castanopsis sclerophylla*	9.19	8.85	20	*Loropetalum chinense*
	5	266	19	Southeast, 116	Evergreen broadleaved forests	0.72	*Schima superba*	9.78	9.86	20	Symplocos sumuntia
	6	261	27	Southeast, 157	Evergreen broadleaved forests	0.60	*Schima superba*	9.68	10.06	20	Indocalamus tessellatus
**LQ-LJ**	7	263	19	Northwest, 303	Coniferous forests	0.65	*Pinus massoniana*	17.51	17.08	30	*Lindera aggregata*
	8	271	24	West, 286	Coniferous forests	0.60	*Pinus massoniana*	18.51	17.41	30	*Lindera aggregata*
	9	287	30	West, 291	Coniferous forests	0.55	*Pinus massoniana*	16.22	14.59	30	*Lindera aggregata*
**CN**	10	194	28	Northeast, 38	Grassland	0	/	/	/	/	*Morella rubra*
	11	201	26	Northwest, 332	Grassland	0	/	/	/	/	*Morella rubra*
	12	202	27	Northeast, 29	Grassland	0	/	/	/	/	*Pittosporum tobira*

LA, Lin’an District in Hangzhou; CA, Chun’an County in Hangzhou; LQ-AR, An’ren Township of Longquan City in Lishui; LQ-LJ, Lanju Township of Longquan City in Lishui; CN, Cangnan County in Wenzhou.

**Table 3:** Herb layer information of study areas.

Study sites	Dominant herb species (importance value/coverage)		
**LA**	*Dicranopteris dichotoma* (51.23/14.67)	*Woodwardia japonica* (17.79/1.27)	*Ophiopogon bodinieri* (11.85/3.00)
**CA**	*Dicranopteris dichotoma* (64.84/48.56)	*Woodwardia japonica* (11.66/9.33)	*Paederia foetida* (8.49/6.33)
**LQ-AR**	*Dicranopteris dichotoma* (57.89/41.55)	*Pteridium aquilinum* (16.50/2.25)	*Woodwardia japonica* (8.86/3.50)
**LQ-LJ**	*Dicranopteris dichotoma* (68.02/92.50)	*Pteridium aquilinum* (17.80/6.42)	*Woodwardia japonica* (4.23/5.00)
**CN**	*Dicranopteris dichotoma* (51.62/96.50)	*Miscanthus floridulus* (31.25/23.33)	*Smilax china* (8.99/2.50)

LA, Lin’an District in Hangzhou; CA, Chun’an County in Hangzhou; LQ-AR, An’ren Township of Longquan City in Lishui; LQ-LJ, Lanju Township of Longquan City in Lishui; CN, Cangnan County in Wenzhou.

We established 15 tree plots (20 m × 20 m) in the five study areas using a completely randomized design (**Figure 1**). The vegetation in the three experimental plots in the same study area was relatively consistent. In each plot, three shrub plots (5 m × 5 m) were established diagonally, and three herbaceous plots (1 m × 1 m) were established along the diagonal in each shrub plot, resulting in a total of 15 tree stands, 45 shrub plots, and 135 herbaceous plots across the five study areas. From July to September 2022, we conducted a field survey of plant characteristics (**Tables 2**, **3**). The occurrence probability of each species in all sample plots was recorded to calculate the relative frequencies.

### 2.2 Plant sampling and measurement

To determine the clonal dispersal traits of *D. dichotoma*, an herbaceous plot (1 m × 1 m) was harvested from each shrub plot, including both the aboveground parts and root sieve elements. The height of the shoot was measured using a tape measure before harvest. The fronds, stripes, and rhizomes of *D. dichotoma* were separated (with the adventitious root mass being negligible and therefore not separated from the rhizomes), dried in an oven at 80°C to reach a constant dry weight, and weighed. Before drying, the total length of rhizome, mean length of the rhizomes internodes, and mean length of roots were measured.

### 2.3 Soil sampling and physicochemical property analysis

Five soil cores, selected by the S-shaped approach, were collected from the 0-cm to 10-cm profiles in each plot using a 5-cm circular soil auger. To ensure consistency, the five soil samples were thoroughly mixed to homogenize each sample. Undisturbed soil samples were collected at the same position in each profile using a cutting ring with a volume of 100 cm^3^ to measure the soil bulk density and soil water content.

The soil water content was determined using the drying method, the soil bulk density was determined using the cutting ring method (Al-Shammary et al., 2018), and the soil pH was determined using the potentiometric method. The soil samples were air-dried after being sieved (with a 2-mm mesh) for nutrient analysis. The soil total carbon content was determined using a carbon and nitrogen analyzer (Muti-N/C 2100, Analytik Jean AG, Germany). The soil total nitrogen content was determined using the semi-micro-Kjeldahl nitrogen method, as well as with a carbon and nitrogen analyzer. The soil total phosphorus content was determined using acid fusion and the molybdenum-antimony colorimetric method with an ultraviolet spectrophotometer (NanoDrop One, Thermo Fisher Scientific, Waltham, MA, USA). All the analytical methods employed in this study were adapted from the soil agrochemical analysis methods described in Lu (2000).

### 2.4 Data analysis

The importance value of trees, shrubs, or herbs was calculated using the following formulas (Fang et al., 2009):


(1) $$\text{I}{\text{V}}_{\text{Trees}}\left(\%\right)=\left(\text{RD}+\text{RC}+\text{RF}\right)/3$$

(2) $$\text{I}{\text{V}}_{\text{Shurbs}+\text{herbs}}\left(\%\right)=\left(\text{RH}+\text{RC}+\text{RF}\right)/3$$

where *RD*, *RC*, *RF*, and *RH* represent the relative density, relative coverage, relative frequency, and relative height, respectively.

To conduct the variance partitioning analysis, parameters such as community type and topographic factor were determined. For community type, the grass slope was set to 1, the coniferous forest was set to 2, the coniferous and broadleaved mixed forest was set to 3, and the evergreen broadleaved forest was set to 4. The slope aspect was transformed into the transformation of the aspect index (TRASP), ranging between 0 and 1, with the following formula:


(3) $$\text{TRASP}=\{1-\text{cos}\left[\left(\frac{\text{π}}{180}\right)\left(\text{aspect}-30\right)\right]\}/2$$

where *TRASP* is the aspect index and *aspect* are the aspect azimuth angle determined by the compass. Through conversion, *TRASP* falls between 0 and 1, where 0 represents the northeast direction and 1 represents the southwest direction (Wang et al., 2017).

### 2.5 Statistical analysis

One-way analysis of variance and least significant difference tests were conducted for multiple comparisons, with statistical significance assessed at *p* < 0.05. Variance partitioning analysis was used to determine the degree to which the environmental factors explain the coverage and important value of *D. dichotoma*, which was conducted using the R package “vegan.” Regression analysis was used to examine the effects of environmental factors on the growth of *D. dichotoma*, while Pearson correlation analysis was employed to investigate the interactions between soil characteristics and *D. dichotoma*. All statistical analyses and mapping were performed in R (version 4.2.0) and Sigmaplot (Systat, version 15.0).

## 3 Results

### 3.1 Single-dominant-species patches of *D. dichotoma* in different study sites

**Figure 2:**
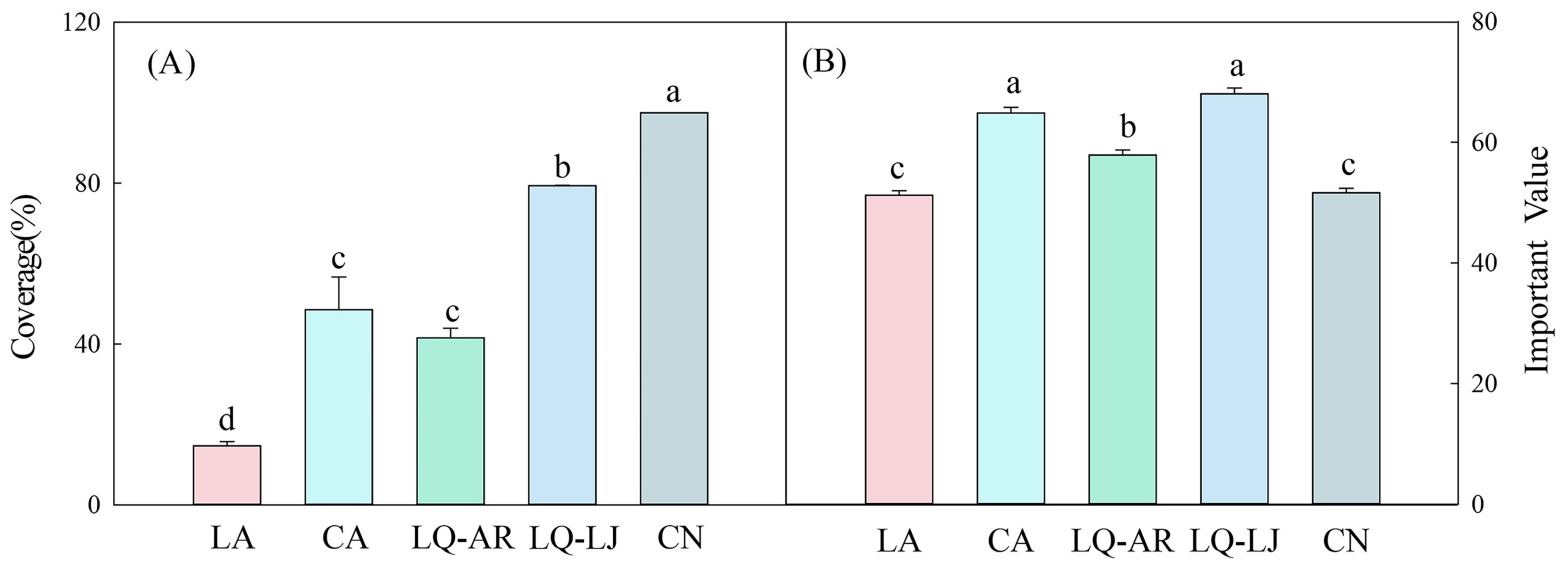
The coverage **(A)** and important value **(B)** of *D. dichotoma* in different study sites. LA, Lin’an District in Hangzhou; CA, Chun’an County in Hangzhou; LQ-AR, An’ren Township of Longquan City in Lishui; LQ-LJ, Lanju Township of Longquan City in Lishui; CN, Cangnan County in Wenzhou. Different lowercase letters represent significant differences among the study sites (*P* < 0.05). Data represent mean ± SD (n=9).

Higher coverage and importance values indicate a stronger capacity for *D. dichotoma* to form single-dominant-species patches. *D. dichotoma* exhibited the highest coverage and importance value in LQ-LJ (**Figure 2**). Although the coverage of *D. dichotoma* in CN was also high, its importance value was the lowest, suggesting the presence of other herbaceous species within the patches. **Table 3** shows that *Miscanthus floridulus* was the dominant associated species in the herb layer in CN. *M. floridulus* is a tall herb that readily forms single-species-dominant patches.

In CA and LQ-AR, the coverage of *D. dichotoma* was approximately 50%, roughly half that observed in LQ-LJ; however, the importance values were relatively higher. The coverage of *D. dichotoma* in LA was the lowest at 14%, which also had the lowest importance value among the sites. Consequently, the formation of single-dominant-species patches of *D. dichotoma* appears to be the most conducive in LQ-LJ, followed by CN, LQ-AR, CA, and is the least conducive in LA.

### 3.2 Clonal dispersal traits of *D. dichotoma* in different study sites

**Figure 3:**
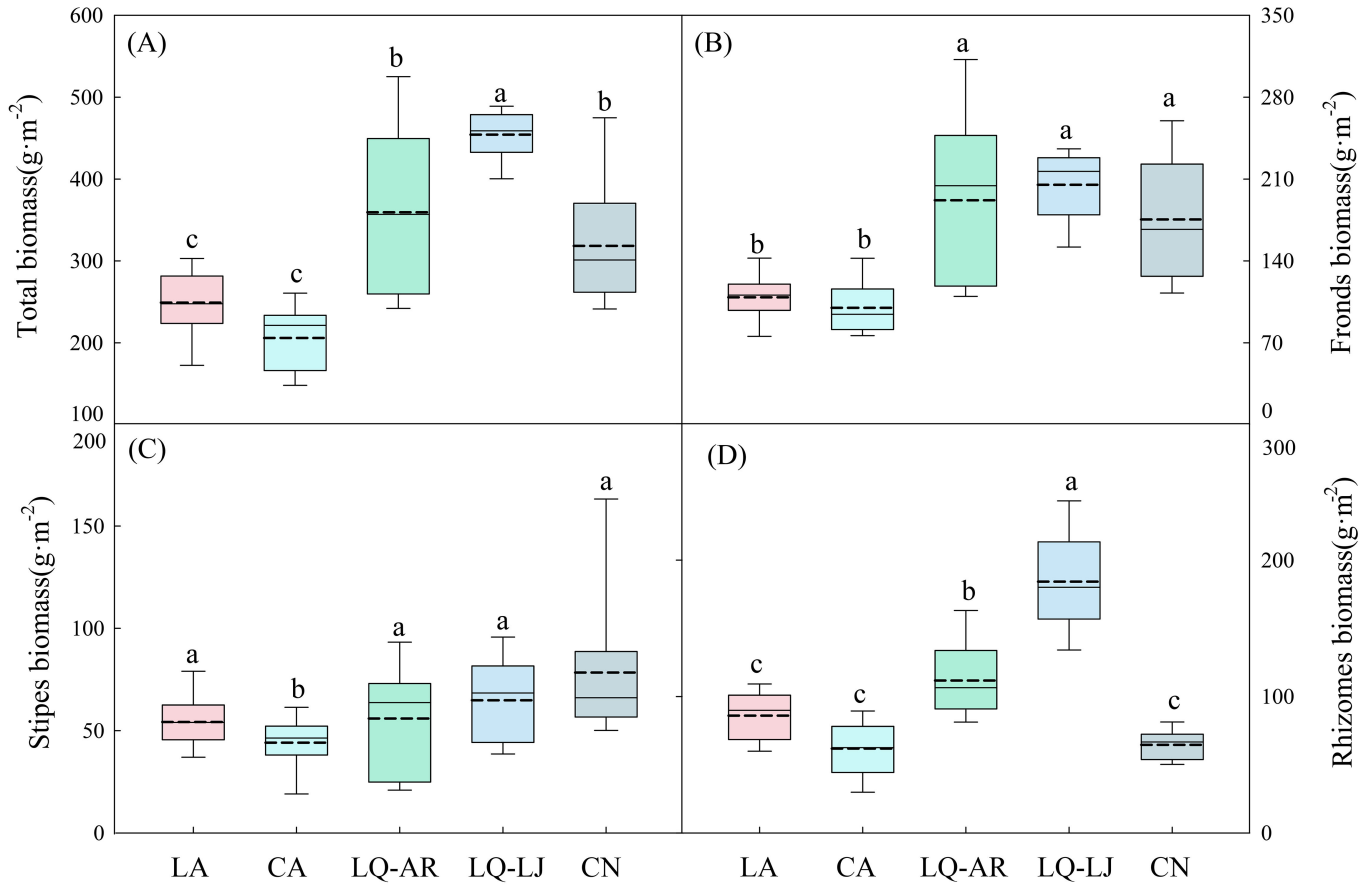
Total biomass **(A)**, fronds biomass **(B)**, stipes biomass **(C)**, and rhizomes biomass **(D)** of *D. dichotoma* in different study sites (*n* = 9). LA, Lin’an District in Hangzhou; CA, Chun’an County in Hangzhou; LQ-AR, An’ren Township of Longquan City in Lishui; LQ-LJ, Lanju Township of Longquan City in Lishui; CN, Cangnan County in Wenzhou. Different lowercase letters represent significant differences among the study sites (*p* < 0.05). The upper and lower boundaries of the box represent the upper quartile and the lower quartile, respectively. The middle solid line and dashed line indicate the median and mean value, respectively. The whisker lines extend to the maximum and minimum values.

**Figure 4:**
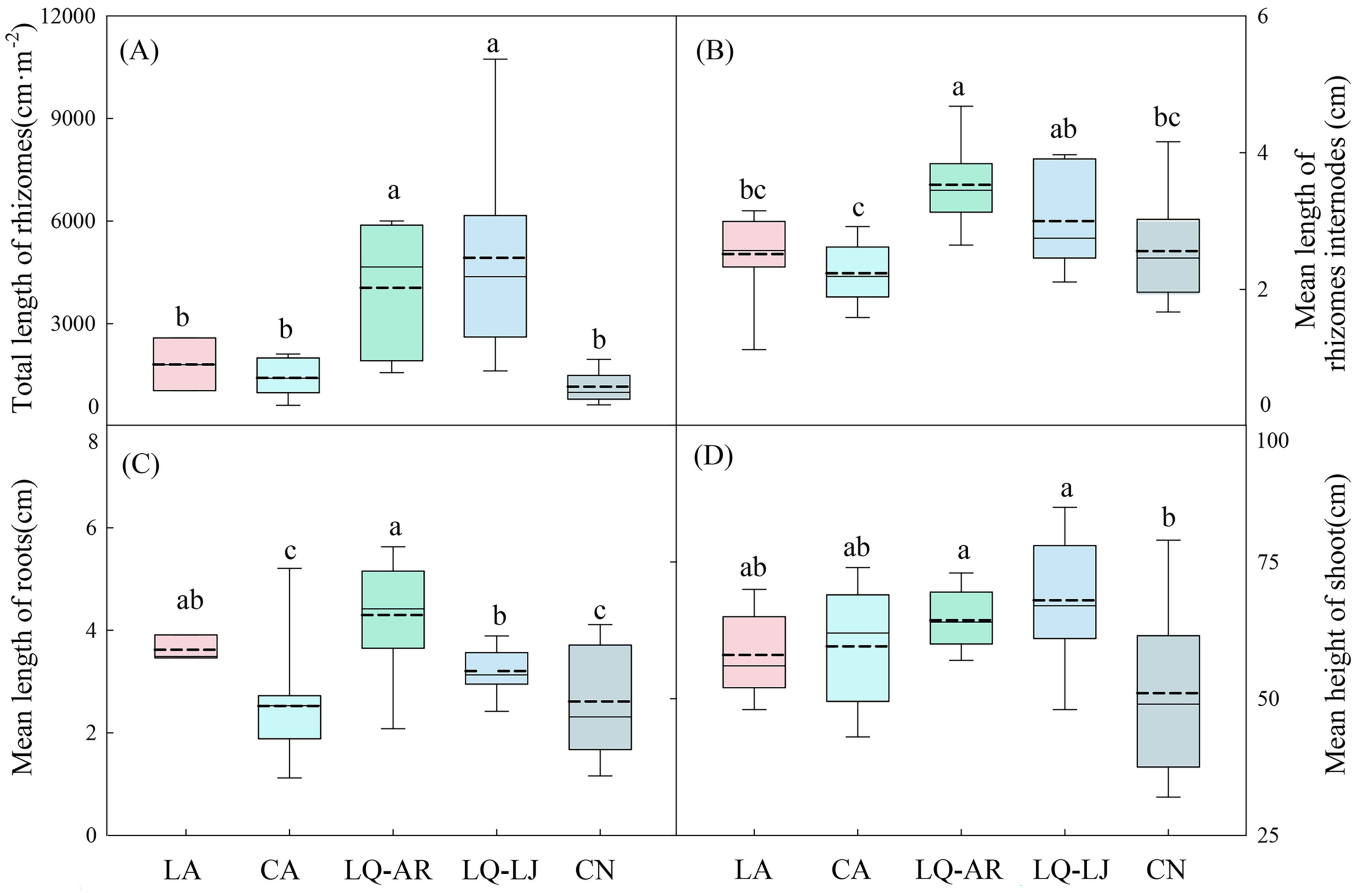
Total length of rhizomes **(A)**, Mean length of rhizomes internodes **(B)**, Mean length of roots **(C)**, and Mean length of shoot **(D)** of *D. dichotoma* in different study sites (*n* = 9). LA, Lin’an District in Hangzhou; CA, Chun’an County in Hangzhou; LQ-AR, An’ren Township of Longquan City in Lishui; LQ-LJ, Lanju Township of Longquan City in Lishui; CN, Cangnan County in Wenzhou. Different lowercase letters represent significant differences among the study sites (*p* < 0.05). The upper and lower boundaries of the box represent the upper quartile and the lower quartile, respectively. The middle solid line and dashed line indicate the median and mean value, respectively. The whisker lines extend to the maximum and minimum values.

The highest biomass of *D. dichotoma* was observed in LQ-LJ (454.27 g·m^−2^), followed by LQ-AR and CN, with the lowest biomass recorded in LA and CA (**Figure 3A**), representing a significant difference (*p* < 0.05). The biomass of fronds in LQ-LJ, LQ-AR, and CN was significantly higher than that in CA and LA (**Figure 3B**, *p* < 0.05), while the biomass of stipes in CA was significantly lower than that in the other four study sites (**Figure 3C**, *p* < 0.05). The biomass of the rhizomes was the greatest in LQ-LJ, indicating that the reproductive ability of *D. dichotoma* is the strongest in that area (**Figure 3D**, *p* < 0.05). The total length of rhizomes and the mean length of rhizome internodes, which reflect the plant’s clonal dispersal ability, were the highest in LQ-LJ and LQ-AR (**Figures 4A, B**, *p* < 0.05). The highest mean root length was observed in LA and LQ-AR, followed by LQ-LJ, while the lowest was in CN and CN (**Figure 4C**, p<0.05). Among the study sites, CN had the lowest mean height of shoots, with the remaining four sites exhibiting no significant differences, averaging approximately 60 cm (**Figure 4D**).

**Figure 5:**
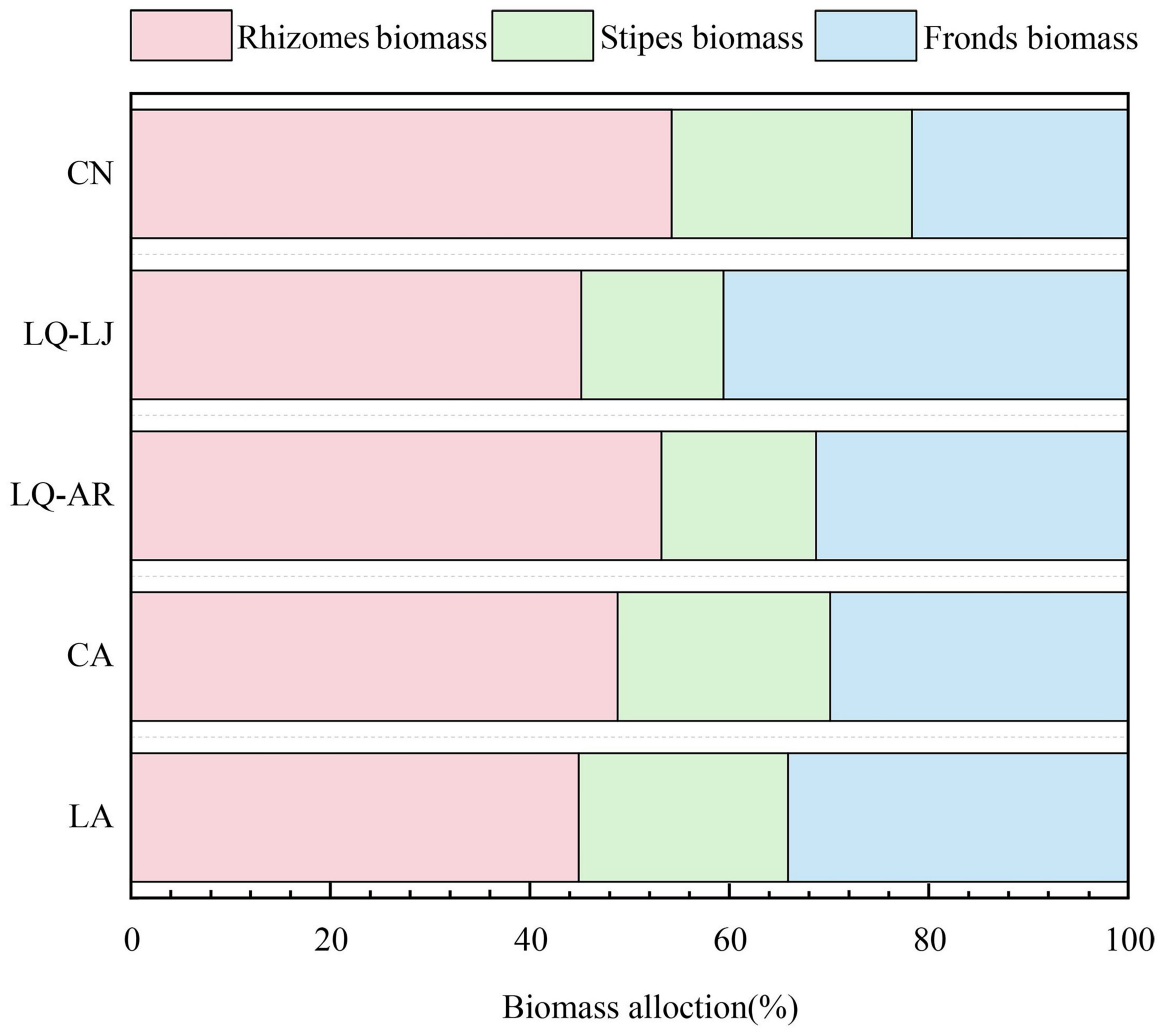
Biomass allocation of *D. dichotoma* in different study sites (*n* = 9). LA, Lin’an District in Hangzhou; CA, Chun’an County in Hangzhou; LQ-AR, An’ren Township of Longquan City in Lishui; LQ-LJ, Lanju Township of Longquan City in Lishui; CN, Cangnan County in Wenzhou.

*D. dichotoma* was found to allocate the majority of its biomass to the fronds and rhizomes, with a smaller portion allocated to the stipes (**Figure 5**). However, there were significant differences in biomass allocation ratios among different study sites. In LQ-AR and LQ-LJ, the biomass allocation ratio of *D. dichotoma* to the stipes was only 14%–16%, while those to fronds and rhizomes reached up to 84%. In other sites, the biomass allocation ratio to the stipes exceeded 21%, with those to the fronds and rhizomes ranging from 76% to 79%.

### 3.3 Effects of climate and topography on single-dominant-species patches of *D. dichotoma*

**Figure 6:**
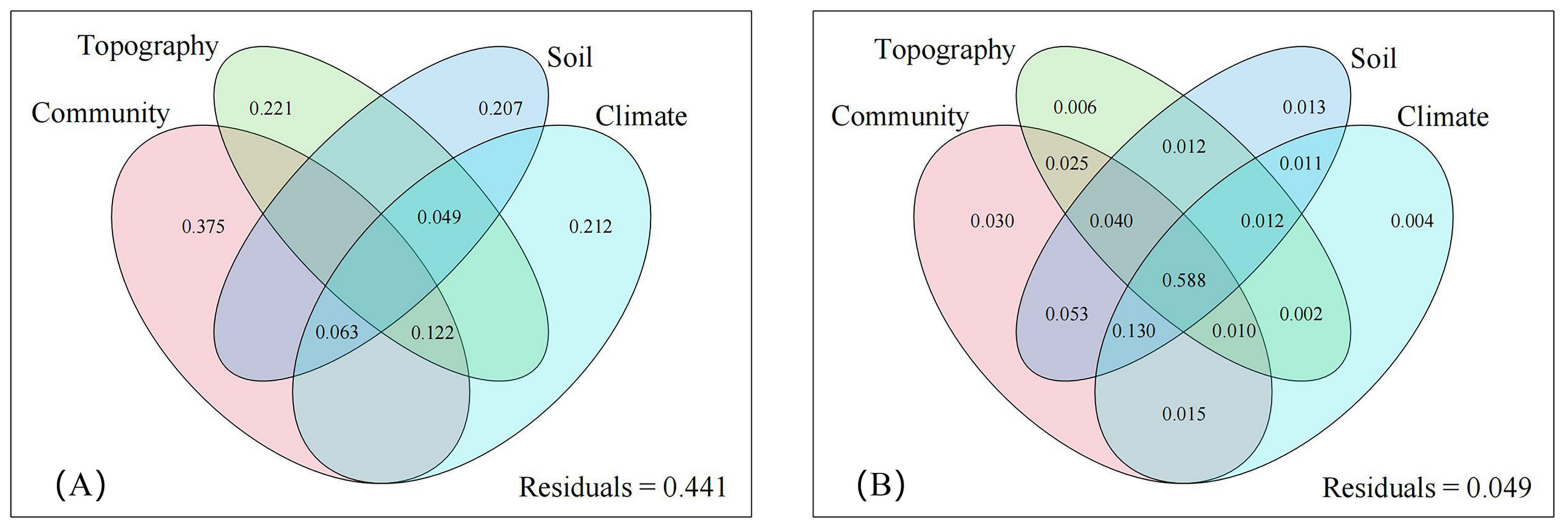
Explanatory of different environmental factors for coverage **(A)** and important value **(B)** of *D*. *dichotoma*. The climate was annual mean temperature, annual mean precipitation, and annual mean sunshine hours. The topography was elevation, slope, and aspect. The community were canopy closure, community type, and understory light intensity. The soil was pH, soil water content, soil bulk density, total carbon content, total nitrogen content, and total phosphorus content.

Climate and topography had a minimal impact on the importance value of *D. dichotoma* but significantly influenced its coverage (**Figure 6**). Topography affected the coverage and importance value of *D. dichotoma* by 22.1% and 0.6%, respectively. **Table 4** shows that the clonal dispersal traits of *D. dichotoma*, which determined the internal factors affecting its coverage, were significantly positively correlated with elevation and significantly negatively correlated with slope. It explains why *D. dichotoma* prefers to grow on gentle slopes below an elevation of 300 m (**Table 2**).

**Table 4:** Correlation of clonal dispersal traits of *D. dichotoma* with climate, community, and topography characteristics.

Clonal dispersal traits	Annual mean temperature	Annual mean precipitation	Annual mean sunshine hours	Elevation	Slope	Aspect	Canopy closure	Community type	Understory light intensity
**Fronds** **Mass**	ns	ns	−0.320*	0.373*	−0.334*	ns	−0.295*	ns	0.393**
**Stipes** **Mass**	ns	ns	ns	ns	−0.335*	−0.332*	−0.373*	−0.375*	ns
**Rhizomes mass**	−0.352*	0.487**	ns	0.752**	Ns	0.401**	ns	ns	ns
**Total length of rhizomes**	−0.364*	0.451**	ns	0.684**	Ns	ns	ns	ns	ns
**Mean length of rhizomes internodes**	ns	ns	ns	−0.456**	Ns	ns	ns	ns	ns
**Mean length of roots**	0.366*	ns	ns	0.435**	ns	ns	ns	0.437**	ns
**Mean height of shoot**	0.349*	ns	0.300*	0.456**	ns	0.307*	ns	ns	ns

*Correlations with *P* < 0.05; **Correlations with *P* < 0.01; ns, non-significant correlations with *P* > 0.05.

**Figure 7:**
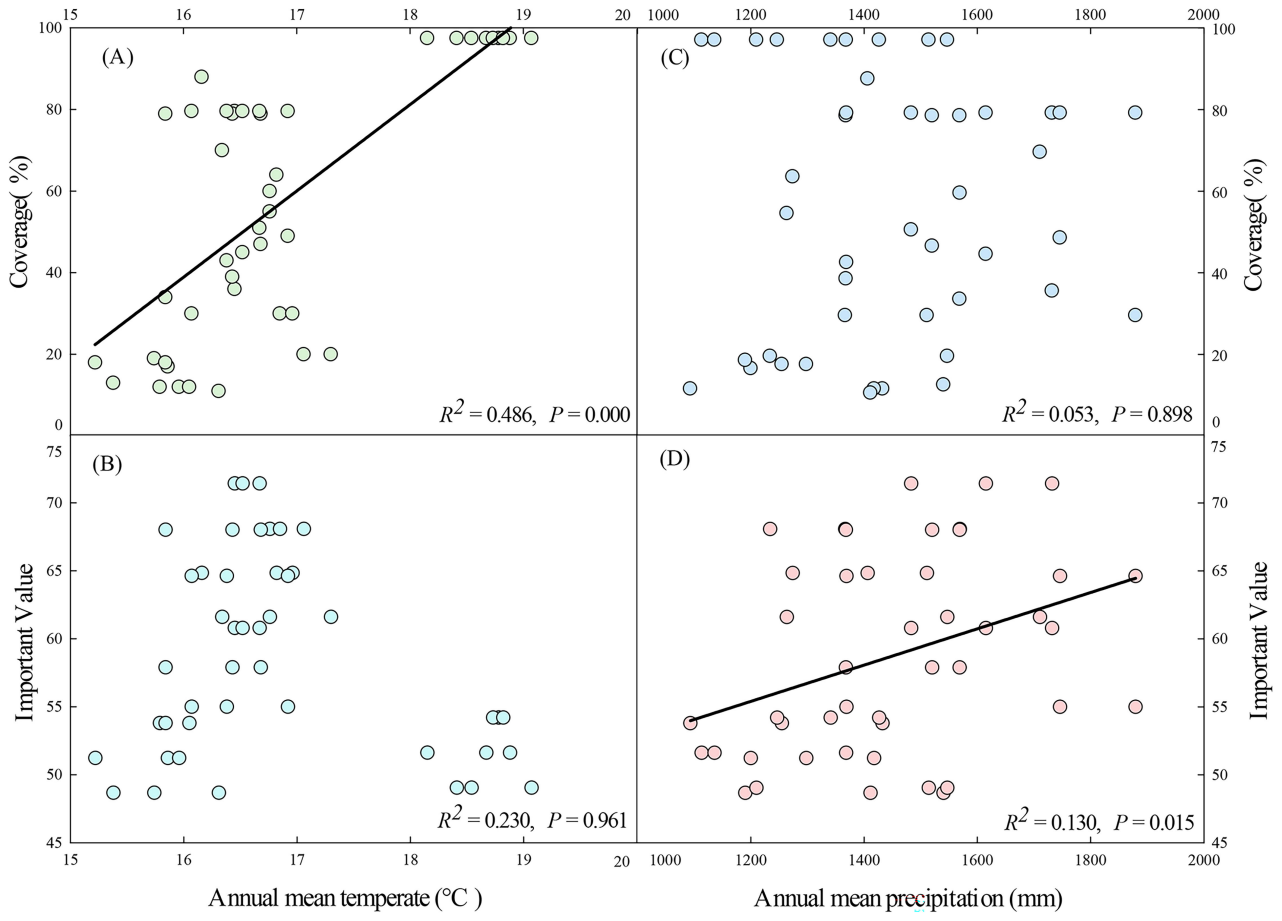
Annual mean temperature in relation to coverage **(A)** and important value **(B)** of D. dichotoma. Annual mean precipitation in relation to coverage **(C)** and important value **(D)** of *D. dichotoma* (n=45). Solid lines represent the fitted linear regression model and indicate significant relationship (*P* < 0.05).

Climate affected both the coverage and importance value of *D. dichotoma* by 21.2% and 0.4%, respectively. The coverage of *D. dichotoma* showed a significant positive correlation with annual mean temperature (r = 0.697, **Figure 7A**), whereas its importance value did not correlate significantly with this temperature variable (**Figure 7B**). The coverage of *D. dichotoma* did not show a significant correlation with annual mean precipitation (**Figure 7C**). However, its importance value had a significant positive correlation with annual mean precipitation, although the correlation coefficient was low at 0.360 (**Figure 7D**). Therefore, climate and topography mainly affected the coverage of *D. dichotoma*, while its impact on the importance value was not significant. In addition, the rhizomes biomass and total length of rhizomes were significantly negatively correlated with the annual mean temperature and significantly positively correlated with the annual mean precipitation (**Table 4**).

### 3.4 Effects of community factors on patches of *D. dichotoma*

The influence of community factors on the coverage of *D. dichotoma* was the greatest among the four environmental factors, reaching up to 37.5% (**Figure 6A**). Community types, canopy closure, and understory light intensity exhibit strong autocorrelation; thus, only the effects of understory light intensity on the formation of single-dominant-species patches of *D. dichotoma* were analyzed.

**Figure 8:**
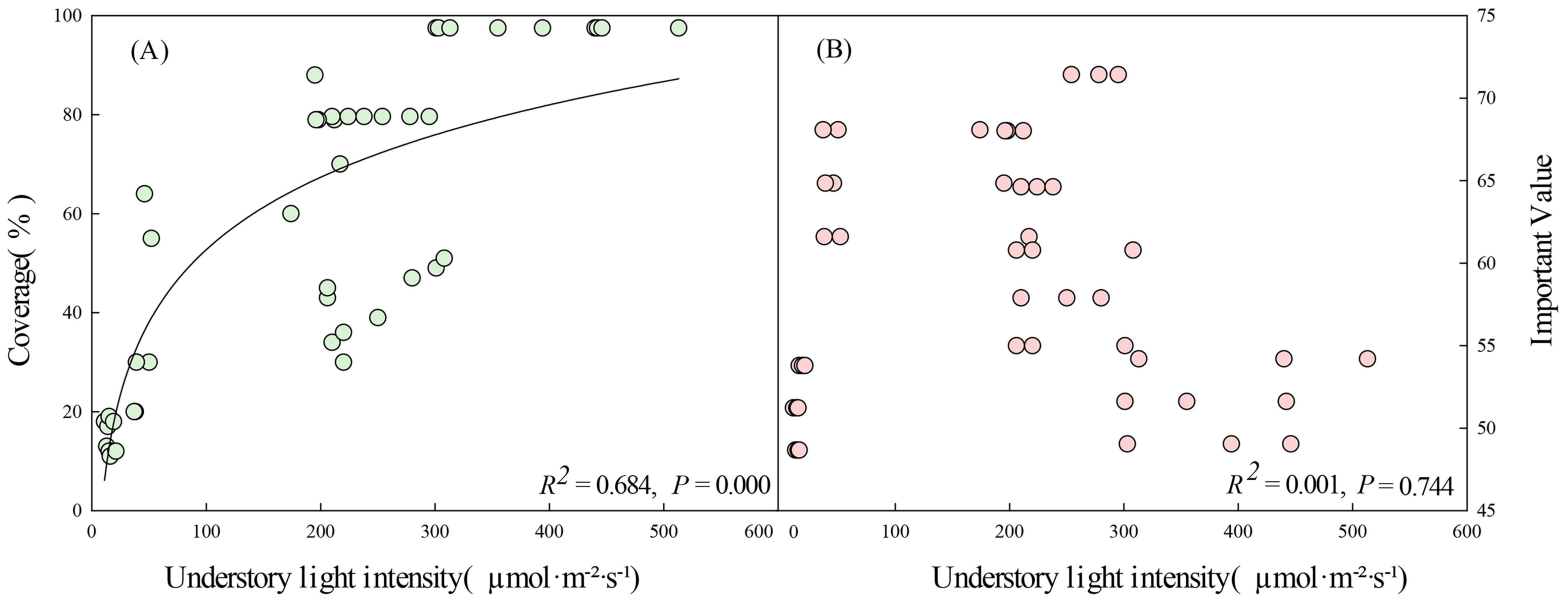
Understory light intensity in relation to coverage **(A)** and important value **(B)** of *D. dichotoma* (n=45). Solid lines represent the fitted linear regression model and indicate a significant relationship (*P* < 0.05).

A logarithmic correlation was found between understory light intensity and coverage of *D. dichotoma*, with a correlation coefficient of 0.827 (**Figure 8A**, *p* < 0.05). The coverage of *D. dichotoma* initially increased rapidly with increasing understory light intensity and then leveled off. At light intensities of 200–400 µmol·m⁻²·s⁻¹, the coverage ranged from 40% to 80%, whereas at light intensities of 400–500 µmol·m⁻²·s⁻¹ the coverage approached 100%.

By contrast, community factors affected the importance value of *D. dichotoma* only by 3.0% (**Figure 6B**), and understory light intensity also did not have a significant impact on the importance value of *D. dichotoma* (**Figure 8B**). Furthermore, the fronds and stipes biomass of *D. dichotoma* was significantly negatively correlated with the community canopy, and significantly positively correlated with the understory light intensity (**Table 4**).

### 3.5 Correlations between soil properties and patches of *D. dichotoma*

**Figure 9:**
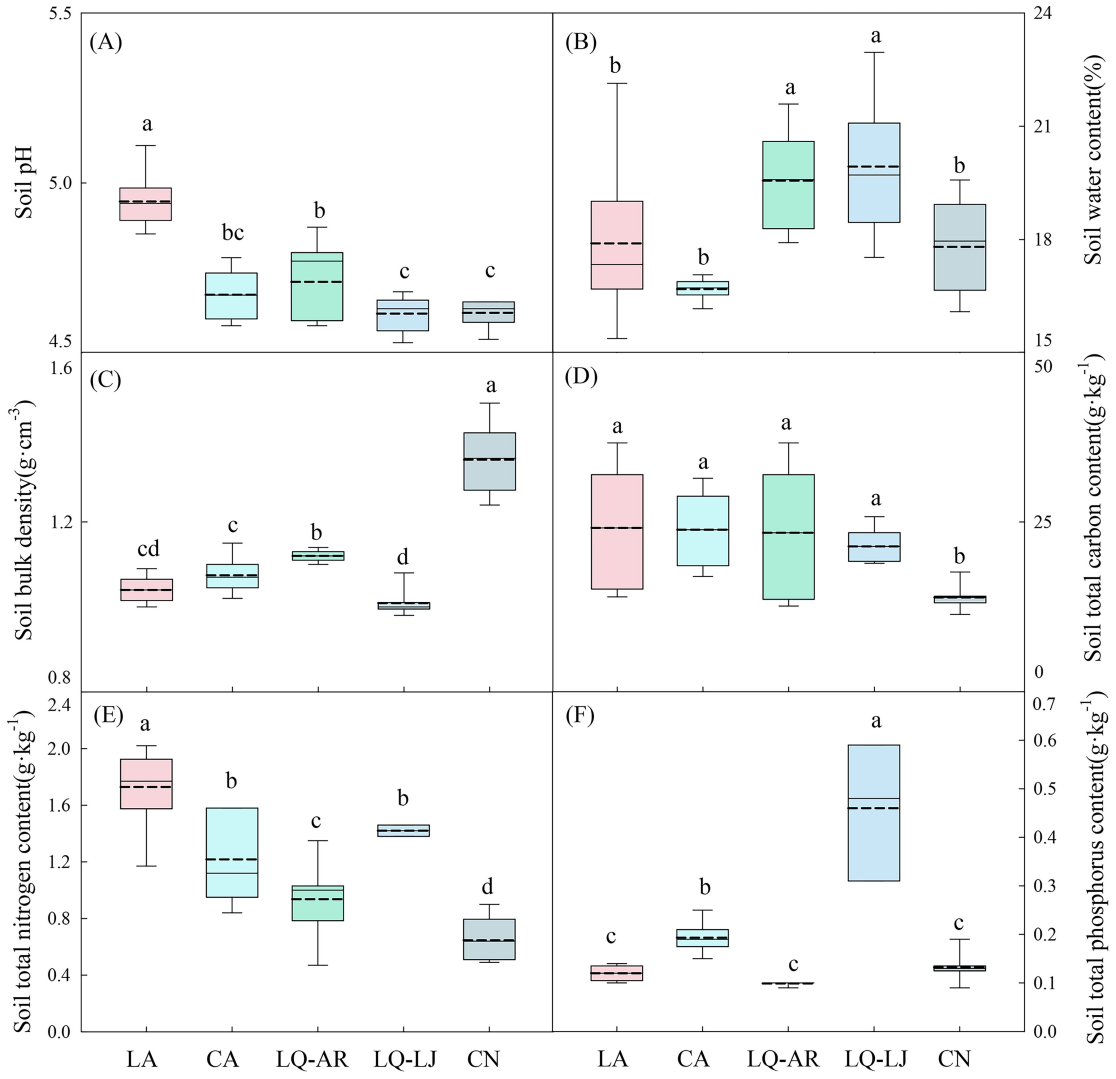
Soil pH **(A)**, soil water content **(B)**, soil bulk density **(C)**, soil total carbon content **(D)**, soil total nitrogen content **(E)**, soil total phosphorus content **(F)** in different study sites (n=9). LA, Lin’an District in Hangzhou; CA, Chun’an County in Hangzhou; LQ-AR, An’ren Township of Longquan City in Lishui; LQ-LJ, Lanju Township of Longquan City in Lishui; CN, Cangnan County in Wenzhou. Different lowercase letters represent significant differences among the study sites (*p* < 0.05). The upper and lower boundaries of the box represent the upper quartile and the lower quartile, respectively. The middle solid line and the dashed line indicate the median and mean value, respectively. The whisker lines extend to the maximum and minimum values.

*D. dichotoma* was mainly found in acidic and moist soil. **Figure 9** shows that the soil pH was in the range of 4.53–5.11, the soil water content was 15.38%–22.96%, and the soil bulk density was 0.98–1.48 g·cm^−3^. *D. dichotoma* exhibits strong adaptability to soil nutrients and was able to grow in soils with very low total carbon, nitrogen, and phosphorus contents (**Figures 9D–F**).

Soil characteristics contributed 20.7% to the cover of *D. dichotoma* and contributed 1.3% to its importance value (**Figure 6**). The clonal dispersal traits of *D. dichotoma* were significantly positively correlated with soil moisture and total phosphorus content and were significantly negatively correlated with soil bulk density (**Table 5**). The coverage of *D. dichotoma* was significantly negatively correlated with soil pH, total carbon, and total nitrogen content and was significantly positively correlated with soil bulk density and total phosphorus content. The importance value of *D. dichotoma* was significantly negatively correlated with soil pH and bulk density and was significantly positively correlated with total nitrogen and total phosphorus contents (**Table 5**). These findings indicated that soil and *D. dichotoma* characteristics can indeed influence each other.

**Table 5:** Correlation of clonal dispersal traits of *D. dichotoma* with soil characteristics.

Clonal dispersal traits	Soil pH	Soil water content	Soil bulk density	Soil total carbon content	Soil total nitrogen content	Soil total phosphorus content
**Coverage**	−0.374*	ns	0.517**	−0.433**	−0.564**	0.354*
**Important value**	−0.400**	ns	−0.498**	ns	ns	0.670**
**Fronds mass**	−0.371*	0.299*	ns	Ns	ns	0.318*
**Stipes mass**	ns	ns	0.415**	Ns	ns	ns
**Rhizomes mass**	ns	0.478**	−0.461**	Ns	ns	0.691**
**Total length of rhizomes**	ns	0.361*	−0.378*	Ns	ns	0.371*
**Mean length of rhizomes internodes**	ns	0.356*	ns	Ns	ns	ns
**Mean length of roots**	ns	0.386*	ns	Ns	ns	ns
**Mean height of shoot**	ns	ns	−0.317*	Ns	ns	0.360*

*Correlations with *P* < 0.05; **correlations with *P* < 0.01; ns, non-significant correlations with *P* > 0.05.

## 4 Discussion

### 4.1 Effect of climate on single-dominant-species patches of *D. dichotoma*

In this study, *D. dichotoma* was typically found on slopes or gentle slopes below an altitude of 300 m. This is consistent with a previous study showing that *D. dichotoma* mainly grew in subtropical low mountains and hills (Zhang et al., 2010a). We found that *D. dichotoma* could form large single-dominant-species patches under coniferous forests, evergreen broadleaved forests, conifer-broadleaf forests, and even in unforested lands. Yang et al. (2021) found that the distribution of *D. dichotoma* primarily stretched from the south of the Yangtze River in China to the Pacific Islands. In summary, at the regional scale, the primary environmental factor influencing the formation of the dominant *D. dichotoma* community appears more likely to be related to climate than community characteristics.

Although *D. dichotoma* is widely distributed south of the Yangtze River Delta, field surveys have found that it more readily forms extensive stands in the southern Zhejiang Province, while it is more sporadically dispersed in the northern Zhejiang Province. Our study provides further evidence that higher annual mean temperature and annual mean precipitation are factors that are more conducive to the formation of single-dominant-species patches of *D. dichotoma*. The mechanism is considered to be due to the fact that high temperature and high humidity promote the clonal growth and clonal dispersal of *D. dichotoma*. Therefore, at a regional scale, climate is the primary driver for the formation of single-dominant-species patches of *D. dichotoma*.

Although higher annual mean precipitation was associated with a larger importance value of the *D. dichotoma* community, the individual contribution of climate to the importance value was not substantial. This can explain the finding that the CN study site had the highest annual mean temperature and annual mean precipitation, but a relatively low importance value of *D. dichotoma*. This is largely because such a hot and humid climate also benefits the growth of other herbaceous plants, including competing species such as *M. floridulus*.

In addition, the individual contributions of climate, topography, community, and soil factor to the importance value of *D. dichotoma* were each very low, their combined effect on the importance value reached 58.8%, indicating that the formation of single-dominant-species patches is determined by the comprehensive interaction of these four environmental factors.

### 4.2 Effects of community factors on single-dominant-species patches of *D. dichotoma*

As a light-demanding fern, understory light intensity is considered to be the dominant factor for the growth of *D. dichotoma* (Zhang et al., 2010b), which was confirmed by our results. Specifically, we found that an understory light intensity of 200–500 µmol·m⁻²·s⁻¹ was the most favorable condition for the formation of the *D. dichotoma* community. This was consistent with the results of our previous controlled pot and semi-controlled field experiments, which showed that *D. dichotoma* does not tolerate strong light (Jin, 2019). This is because strong light and high temperatures will damage the donor side, acceptor side, and reaction center of photosystem II. Additionally, these conditions can lead to the degradation of pigment proteins in the leaves, resulting in a reduction in the photosynthetic capacity (Liao et al., 2023).

Community characteristics have minimal and non-significant effects on the rhizomes biomass and total length of rhizomes, but significantly influenced the biomass allocation of *D. dichotoma*. In communities with an understory light intensity of 200–300 µmol·m⁻²·s⁻¹, *D. dichotoma* exhibited the best development of dominant layers, with the lowest biomass investment in the supporting structures. This enables *D. dichotoma* to allocate more resources to the fronds or rhizomes, thereby enhancing its productivity and reproductive capacity (Liu et al., 2008). Therefore, at the community scale, variations in understory light intensity alter biomass investment in different clonal modulars, thereby influencing the formation of single dominant populations of *D. dichotoma*.

Our previous studies revealed that *D. dichotoma* had a greater light saturation point and a lower light compensation point than those of many other species (Liao et al., 2023). Yu et al. (2023) demonstrated that *D. dichotoma* adapted to low-light environments in evergreen broadleaved forests by increasing its specific leaf area, soil total nitrogen, soil total phosphorus, and chlorophyll content in the fronds. The disappearance of *D. dichotoma* in evergreen broadleaved forests could potentially be linked to a shift in resource allocation. Specifically, more resources tended to be allocated to the fronds (e.g., nitrogen and phosphorus), with a compensatory reduction in the allocation of resources to the reproductive organs. The growth and sprouting of rhizomes is a vital pathway for the rapid expansion of *D. dichotoma* (Liu, 2017). Furthermore, some studies have reported that mycorrhization in the ferns was bound to light intensity, with fungal hyphae extending across the root surface area of ferns and improving nutrient absorption (Lehnert et al., 2017; Deng et al., 2023; Li et al., 2023). A substantial decrease in the arbuscular mycorrhizal fungi richness and abundance in the roots of *Struthiopteris spicant* (L.) Weiss, a terrestrial fern distributed in northwestern North America and across Europe, has been reported when they are grown under low light conditions (Molino et al., 2019; Guillen-Otero et al., 2024). Simultaneously, high temperatures and strong light stress are less frequent in evergreen broadleaved forests than coniferous forests. More favorable growth conditions for plant species and increased interspecies competition for *D. dichotoma* are therefore common in evergreen broadleaved forests (Pang et al., 2018). Consequently, restricted rhizome growth and increased interspecific competition might significantly inhibit the growth of *D. dichotoma* in forests with low understory light.

### 4.3 Interaction between soil characteristics and *D. dichotoma*

Soil is a crucial ecological factor influencing the growth of *D. dichotoma*, which favors acidic red-yellow soil with low pH. However, the growth of *D. dichotoma* also significantly impacts soil characteristics. Previous studies showed that *D. dichotoma* can further lower the soil pH by secreting organic acids and other substances (Liu et al., 2012). The present study confirmed a significant negative correlation between soil pH and coverage of *D. dichotoma*, validating the reciprocal influence between soil properties and *D. dichotoma*. Additionally, we discovered that lower soil total carbon and total nitrogen contents were associated with higher *D. dichotoma* coverage. This was attributed to the ability of *D. dichotoma* to tolerate nutrient-poor soil where high-nutrient-demand herbaceous plants cannot thrive.

Previous studies proposed that although *D. dichotoma* can tolerate nutrient-poor soil, it exhibits better growth in loose and fertile soil. However, the specific soil nutrients that limit the formation of a *D. dichotoma* community have not been determined. Our correlation analysis between clonal dispersal characteristics of *D. dichotoma* and soil properties indicated a preference for moist and loose soil with a high phosphorus content. This finding further confirms that soil total phosphorus is a limiting factor for the formation of single-dominant-species patches of *D. dichotoma*. The subtropical region of China is a phosphorus-deprived area, with a soil total phosphorus content ranging between 0.1 and 0.4 g·kg^−1^, which is lower than that of the red soil in southern China at 0.56 g·kg^−1^ (Fang et al., 2018). Moreover, the predominant acidic red soil in this region, which is rich in iron and aluminum oxides, strongly adsorbs and fixes phosphorus, resulting in an extremely low effective phosphorus content. However, the specific mechanisms underlying these effects require further in-depth research.

## 5 Conclusion

Climate was identified as the primary factor influencing the formation of single-dominant-species patches of *D. dichotoma* at the regional scale. Higher annual mean temperature and annual mean precipitation were associated with an increased coverage and increased importance value of *D. dichotoma*. At the community scale, community characteristics affected the formation of *D. dichotoma*-dominant patches. Understory light intensities in the range of 200–500 µmol·m⁻²·s⁻¹ were found to be most suitable for the development of large *D. dichotoma* patches. Soil total phosphorus content emerged as a limiting factor for the development of the *D. dichotoma* community, and the growth of *D. dichotoma* further decreased the soil pH.

## Data Availability

The original contributions presented in the study are included in the article/supplementary material. Further inquiries can be directed to the corresponding author.
